# Supplementary material for: Development and Implementation of a Novel Approach to Scaling the Meeting Centre Intervention for People Living with Dementia and Their Unpaid Carers, Using an Adapted Version of the Template for Intervention Description and Replication (TIDieR) Checklist
Source: Behav Sci (Basel). 2025 May 14;15(5):670. doi: 10.3390/bs15050670 (PMC12109530; doi:10.3390/bs15050670)
Supplement: Supplementary file 1 [file behavsci-15-00670-s001.zip › behavsci-3468895-supplementary.pdf]

Supplementary File S1

Table S1. Stakeholders, their expertise, and their role in Worcestershire Meeting Centre Program.

Stakeholder	Expertise	Description of Role in WMCP
Worcestershire County Council	Senior representative of Communities Directorate	**Funder**: Provided seed funding for the program and technical support at a strategic level in the program's design and implementation such as time frames, vision of scale, and allocation of resources. Was not involved with the day-to-day delivery of the program.
University of Worcester	Program manager<br><br>Program administrator/finance officer<br><br>Research assistant<br><br>Lecturer	**Leader**: Primary organisation responsible for design, implementation, and evaluation of program. Has oversight on strategic and day-to-day decision-making, as well as the development and distribution of program materials and components such as funding awards, training, monitoring, and evaluation.<br>Utilises their independence from health and social care markets and professional reputation to enable interactions within and between services and systems to drive scaling. For example, identifying, communicating, and facilitating opportunities for collaboration and mobilising internal and external resources.
Public Health	Public health practitioner	**Intermediary**: Holds no direct accountability for the program; however, can offer strategic-level technical support from a public health perspective. For example, to inform program design and implementation (e.g. reach, equity of access) by utilising public health data. Uses their authority in relevant professional spaces to share program information and influence implementation outcomes (e.g. penetration, adoption).
Integrated Care System	Dementia lead	**Intermediary**: Holds no direct accountability for the program; however, can offer strategic level technical support for program implementation through mobilising resources, championing vision of program, and enabling channels of

		communication within and between health and social care services.
Services operating as part of the healthcare pathway	Primary care services (General Practice)<br><br>Secondary care services (Memory Assessment Services)<br><br>Third sector post-diagnostic support services	**Intermediary**: Holds no direct accountability for the program; however, can support the implementation of the program through providing information and advice about MCs, and signposting and directly referring people living with dementia (i.e. reach, uptake).
Meeting Centre Providers	Meeting Centre manager<br>Meeting Centre staff<br>Volunteers	**Provider**: Is accountable for contractual obligations; principally, delivering the intervention or integrating the intervention into their existing activities according to the Essential Features of a Meeting Centre.<br>**Funder**: Works to secure funding and investment to ensure sustainability of Meeting Centre.
People living with dementia and their unpaid carers	People living with early to moderate stage dementia and their unpaid carers such as family and friends	**Users**: Individuals who pay a membership fee to utilise the intervention. Should have some level of ownership and input into the operation of the Meeting Centre(s).
